# Lesion-specific comparison of pelvic venous ultrasound, dynamic magnetic resonance venography, and digital subtraction venography in pelvic vein disorders

**DOI:** 10.1016/j.jvsv.2026.102517

**Published:** 2026-05-08

**Authors:** Mayara Leite Coutinho, Lucas Monteiro Delgado, Vinicius Bertoldi, Andrei Skromov de Albuquerque, Joseph Elia Benabou, Marcos Messina, Walter Campos Junior, Nelson de Luccia, Pedro Puech Leão, Antonio Eduardo Zerati

**Affiliations:** a Department of Vascular Surgery, Hospital das Clínicas HCFMUSP, Faculdade de Medicina, Universidade de São Paulo, São Paulo, Brazil; b Universidade Federal de Minas Gerais, Minas Gerais, Brazil; c Department of Radiology, Hospital das Clínicas HCFMUSP, Faculdade de Medicina, Universidade de São Paulo, São Paulo, Brazil; d Department of Obstetrics and Gynecology, Hospital das Clínicas HCFMUSP, Faculdade de Medicina, Universidade de São Paulo, São Paulo, Brazil

**Keywords:** Diagnostic accuracy, Digital subtraction venography, Doppler ultrasound, Dynamic magnetic resonance venography, Gonadal reflux, Iliac vein stenosis, Pelvic vein disorders

## Abstract

**Objective:**

The aim of this study was to compare the diagnostic performance of venous Doppler ultrasound (DUS) and dynamic venous magnetic resonance angiography (dMRV) with digital subtraction venography (DSV) venography in the assessment of anatomical lesions associated with pelvic vein disorders.

**Methods:**

Forty women with symptoms of pelvic vein disorders indicated for endovascular surgical treatment were included in this study. Patients were investigated using DUS and venous dMRV to assess gonadal vein reflux, internal iliac vein reflux, left renal vein (LRV) stenosis, and left common iliac vein (LCIV) stenosis. Intraoperative phlebography images, considered the gold standard, were compared with DUS and magnetic resonance imaging to assess the level of agreement using Cohen’s kappa coefficient. Sensitivity, specificity, predictive values, and overall accuracy were calculated for DUS and dMRV.

**Results:**

No significant agreement was identified between either DUS or dMRV and DSV for grading LRV stenosis (κ = 0.031; *P*-value = .736 for DUS; and κ = 0.146; *P*-value = .151 for dMRV). For LCIV stenosis, DUS showed moderate agreement, whereas dMRV demonstrated no meaningful concordance (κ = 0.490; *P* < .001 for DUS; and κ = 0.211; *P* = .056 for dMRV). In contrast, substantial to excellent agreement with DSV was observed for gonadal vein reflux using dMRV (κ = 0.806; *P* < .001) and moderate agreement using DUS (κ = 0.500; *P* < .001). Neither modality reliably assessed internal iliac vein reflux. Direct comparison between DUS and dMRV showed moderate agreement for gonadal reflux and LRV stenosis, and fair agreement for LCIV stenosis. For detecting gonadal reflux, DUS demonstrated a sensitivity of 100% and dMRV a sensitivity of 93.3%, both with high specificity. Sensitivity for internal iliac vein reflux was low, although specificity remained high for both modalities.

**Conclusions:**

DUS and dMRV demonstrated strong concordance with DSV in detecting gonadal vein reflux. DUS and dMRV are suboptimal imaging modalities in the evaluation of LRV and LCIV stenosis. Both DUS and dMRV are unreliable in the detection of internal iliac vein reflux.

**Article Highlights**

• **Type of Research:** Single-center, cross-sectional, observational study
• **Key Findings:** Forty women with suspected pelvic vein disorders underwent Doppler ultrasound, dynamic magnetic resonance venography, and digital subtraction venography (DSV). Gonadal vein reflux showed strong agreement with DSV using dynamic magnetic resonance venography and moderate agreement using ultrasound. Agreement for renal, iliac, and internal iliac vein disease was limited.
• **Take Home Message:** Noninvasive imaging reliably detects gonadal vein reflux, but DSV remains necessary for comprehensive assessment.

Pelvic vein disorders (PeVDs) are characterized by the presence of pelvic varices, which may be triggered by primary reflux of the gonadal and/or iliac veins, as well as by compression of the left common iliac vein (LCIV) or compression of the left renal vein (LRV) by the aortomesenteric angle.^1^, ^2^, ^3^ It can cause limiting symptoms in affected patients, such as chronic pelvic pain persisting for more than 6 months, worsening after long periods standing, pain during sexual intercourse, and more intense menstrual cramps, as well as urinary urgency and hematuria.^4^^,^^5^

Diagnosis is made through imaging examinations such as abdominal Doppler ultrasound (DUS), abdominal computed tomography angiography, abdominal dynamic venous magnetic resonance angiography (dMRV), and digital subtraction venography (DSV).^6^^,^^7^ Currently, DSV is considered the gold standard for diagnosis, as it allows anatomic assessment of the pelvic venous plexus and the involved veins and, especially, hemodynamic analysis, including intravascular pressure measurements.^8^ It can identify compressive effects, reflux, flow diversion to collateral vessels, and prolonged contrast emptying time—findings that help confirm the presence of PeVD. However, it is an invasive method that uses significant doses of radiation and iodinated contrast.^1^^,^^9^

Given the invasive nature of DSV and the cumulative exposure to radiation and iodinated contrast, there is a growing interest in less-invasive imaging modalities.^6^ In this context, our study was conducted in a pilot outpatient clinic specifically dedicated to PeVD at the Hospital das Clínicas da Faculdade de Medicina da Universidade de São Paulo. The primary objective of this study was to compare the diagnostic accuracy of venous DUS and dMRV with DSV for the assessment of PeVD.

## Methods

### Study design and sample size

This is a cross-sectional study with women followed at the outpatient clinic of the Hospital das Clínicas of the Faculty of Medicine of the University of São Paulo, focused on the diagnosis and treatment of PeVD. As a routine, these women are evaluated using the Numerical Rating Scale for grading pain symptoms and are investigated with abdominal DUS and dMRV. For patients with symptoms of intense pain and radiologic signs of PeVD, surgical intervention is offered, consisting of DSV for diagnostic confirmation and treatment by endovascular technique, depending on the anomaly found. As this was a pilot study aimed at assessing feasibility and generating preliminary agreement estimates, we used a convenience sample; accordingly, no a priori sample size calculation was performed.

### Digital subtraction venography protocol

**Fig 1:**
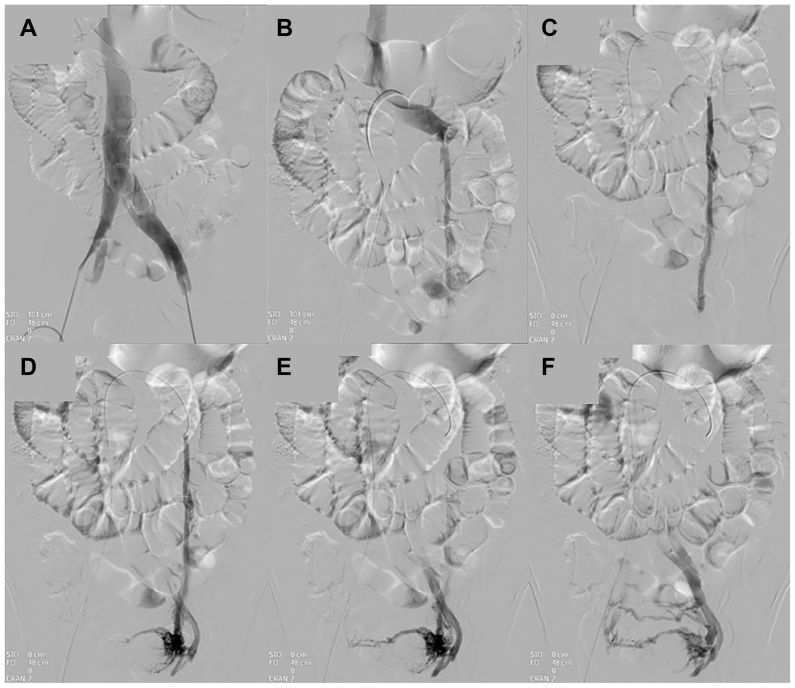
Conventional digital subtraction venography (DSV) obtained through left pedal venous access demonstrating progressive opacification of the pelvic venous system. **(A-F)** Sequential images illustrate contrast reflux into the left gonadal vein with filling of pelvic venous plexuses and collateral pathways, allowing dynamic assessment of venous reflux and pelvic venous congestion. Footnote: The blurred area in the upper left corner corresponds to masking of personal patient data for anonymization purposes.

For the radiologic diagnosis of PeVD, we used the following protocol: DSV was used as the reference standard to evaluate LRV compression between the superior mesenteric artery and the aorta by assessing the caliber difference between the compression point and the prestenotic segment, as well as delayed contrast washout, and by measuring an LRV–inferior vena cava (IVC) pressure gradient ≥3 mmHg. Venous pressure measurements and Valsalva maneuvers were systematically performed in all patients during DSV to assess the dynamic nature of venous compression and to differentiate fixed from positional stenosis. We also assessed reflux into the left gonadal vein, lumbar veins, and periureteral/peripelvic plexuses (Fig 1). For the LCIV, DSV assessed extrinsic compression by the right common iliac artery against the vertebral body, prestenotic dilatation, pelvic/lumbar collaterals, and delayed contrast washout.

### Magnetic resonance angiography protocol

**Fig 2:**
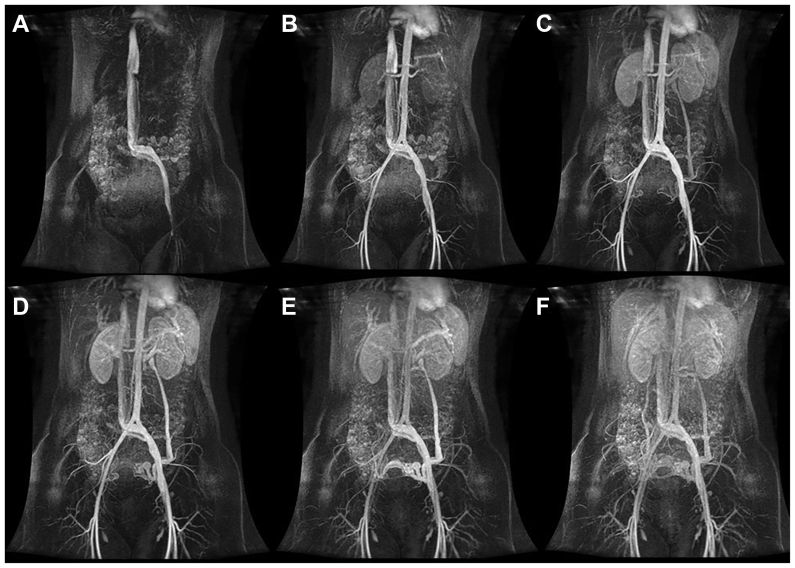
Time-resolved contrast-enhanced dynamic veinous magnetic resonance angiography (dMRV) of the abdomen and pelvis. **(A-F)** Sequential coronal images acquired during dMRV demonstrate progressive venous filling with retrograde opacification of the gonadal vein and pelvic venous plexuses, enabling visualization of reflux pathways and venous congestion.

dMRV was performed using a 1.5-T magnetic resonance imaging scanner (GE Medical Systems, Signa HDxt), following an institutional multiparametric dMRV protocol. The examination included: (1) acquisition of anatomic pelvic images; (2) flow quantification at the distal portion of the common iliac veins; (3) single injection of a gadolinium-based paramagnetic contrast agent through the left pedal vein; (4) time-resolved dynamic contrast-enhanced angiographic imaging of the abdomen and pelvis during contrast injection; and (5) static postcontrast angiographic imaging of the abdomen and pelvis. The time-resolved technique enabled acquisition of multiple angiographic images at short temporal intervals, allowing dynamic assessment of venous return through the left iliac territory, identification of venous obstruction, and detection of flow deviation, closely mimicking invasive DSV (Fig 2). Pedal venous access was used to optimize retrograde opacification of the pelvic venous plexuses and improve visualization of reflux pathways, minimizing early central venous dilution compared with upper-extremity injection.

After arrival of the contrast bolus to the heart, arterial distribution and systemic venous return were also evaluated, allowing assessment of retrograde opacification of the LRV and detection of aortomesenteric compression.

Anatomical pelvic imaging was obtained using a three-dimensional fast spin-echo T2-weighted sequence acquired in the coronal plane, allowing multiplanar reconstructions and detailed evaluation of pelvic organs, pelvic varices, and alternative diagnoses. Pelvic varices were defined as tubular venous structures with a transverse diameter ≥5 mm, typically demonstrating flow voids or high signal intensity in cases of markedly slow flow.

Static anatomic angiographic images were additionally acquired using volumetric T1-weighted gradient-echo and steady-state free precession sequences in the coronal plane, providing high spatial resolution for detailed vascular assessment and accurate measurements.

dMRV assessed gonadal reflux and hypogastric reflux, defined as pathologic retrograde venous flow originating from the internal iliac vein (hypogastric vein) or, more commonly, from its tributary branches, with retrograde drainage into the pelvic venous plexuses resulting in pelvic venous congestion,^8^ including internal spermatic (ovarian), obturator, and accessory internal iliac vein pathways. LRV and LCIV stenosis was graded based on cross-sectional area reduction at the stenotic point relative to the prestenotic segment as mild (<50%), moderate (≥50% and <70%), or severe (≥70%), with significant luminal reduction defined as moderate or severe stenosis.

Anatomic and angiographic images were reviewed using Vue PACS software (Philips, version 12.2), and flow quantification was performed using Cardiac VX software (GE AW VolumeShare 7 workstation).

### Venous Doppler ultrasound protocol

DUS similarly evaluated gonadal and hypogastric reflux (including internal spermatic [ovarian], obturator, and accessory internal iliac vein pathways) during Valsalva maneuver and graded LRV and LCIV stenosis using the hilar-to-compressed diameter ratio (<3, 3-5, >5 for mild, moderate, and severe stenosis, respectively) and the peak velocity ratio between the stenotic and prestenotic segments (<2.0, 2.0-2.5, >2.5 for mild, moderate, and severe stenosis, respectively), aligned with <50%, 50% to 70%, and >70% stenosis categories. All DUS examinations were performed with patients in the supine position and were conducted by an experienced vascular surgeon with expertise in pelvic venous imaging. The same diagnostic protocol was applied to all patients, and each examination was performed independently of the others. Diagnostic criteria for each imaging modality are summarized in Supplementary Table I (online only).

### Eligibility criteria

Women aged 18 years or older were eligible if they were referred to our service with a clinical presentation consistent with PeVD, characterized by chronic pelvic pain with worsening in the orthostatic position and limitation of daily activities, frequently associated with dyspareunia and dysmenorrhea. All symptoms had an overall duration longer than 6 months. Patients were initially referred with prior transvaginal ultrasound demonstrating pelvic varices suggestive of pelvic venous disease.

After referral, the diagnosis was reassessed and confirmed at our institution using additional imaging examinations, including venous DUS and cross-sectional imaging with dMRV, according to clinical indication and availability. Patients with imaging findings consistent with pelvic venous reflux and/or venous obstruction were subsequently referred for intraoperative DSV performed as part of planned endovascular treatment. Only patients who underwent DSV and received endovascular intervention with confirmation of pelvic varices were included in the study.

All imaging examinations were performed within a maximum interval of 2 months, measured from the first noninvasive imaging examination to DSV.

Patients were excluded if they had genital malignancy, acute pelvic inflammatory disease, known allergy to iodinated or gadolinium-based contrast agents, renal insufficiency, a history of prior pelvic radiotherapy for malignancy, previous IVC or iliofemoral deep vein thrombosis, or lower limb symptoms suggestive of primary iliac or IVC obstruction.

### Data collection

Data were collected through retrospective review of the patients’ medical records. We manually extracted demographic and clinical variables, including age, sex, body mass index, American Society of Anesthesiologists physical status, multiparity, and symptom profile (presence and severity of chronic pelvic pain, dyspareunia, and dysmenorrhea). In addition, we evaluated the findings from preoperative imaging exams (DUS, magnetic resonance imaging), including reflux in the gonadal and iliac veins, signs of LRV and left iliac vein compression, and the presence of pelvic varices, using the criteria described above, and compared them with intraoperative phlebography images.

### Ethical approval

The research proposal was reviewed and approved by the Committee of Medical Ethics of the University of São Paulo.

### Statistical analysis

The medical data were recorded on standardized case-report forms and analyzed using SPSS for Windows, version 25.0 (IBM). Continuous variables were expressed as mean and standard deviation and categorical variables as counts and percentages. Venous anatomy and incompetence grades on each imaging modality were summarized as percentages. Agreement between the noninvasive modalities and diagnostic DSV in detecting refluxing pelvic veins was assessed with Cohen’s kappa coefficient, with values closer to 1 indicating stronger agreement.^10^ For each noninvasive method, sensitivity, specificity, positive predictive value, negative predictive value, and overall diagnostic accuracy (proportion of concordant pairs) were calculated using DSV as the reference standard; these accuracy measures are also presented as bar charts. Categorical variables were compared using the χ^2^ test. A two-sided *P* value < .05 was considered statistically significant.

## Results

### Patient characteristics

Forty women with clinical features compatible with PeVD were included. Baseline characteristics are summarized in Table I. The mean age was 39.2 ± 8.0 years, and the mean body mass index was 24.8 ± 2.1 kg/m^2^. Patients were predominantly multiparous, with a mean of 2.4 ± 1.2 pregnancies. The severity of symptoms was high, with mean Numerical Rating Scale scores of 8.9 ± 1.2 for chronic pelvic pain, 9.0 ± 1.4 for dyspareunia, and 8.0 ± 2.4 for dysmenorrhea. Most patients were classified as American Society of Anesthesiologists physical status I (87.5%), with a minority in classes II (10.0%) and III (2.5%).

**Table 1:** Baseline characteristics of the study population (N = 40)

Characteristics	Data
Age, years	39.2 ± 8.0
Body mass index, kg/m^2^	24.8 ± 2.1
No. pregnancies	2.4 ± 1.2
Chronic pelvic pain intensity, NRS	8.9 ± 1.2
Dyspareunia intensity, NRS	9.0 ± 1.4
Dysmenorrhea intensity, NRS	8.0 ± 2.4
ASA I	35 (87.5)
ASA II	4 (10.0)
ASA III	1 (2.5)

*ASA*, American Society of Anesthesiologists physical status classification; *NRS*, Numeric Rating Scale.

Data are presented as mean ± standard deviation or number (%).

### Diagnostic analysis

#### Analysis for left renal vein stenosis

Agreement between imaging modalities and DSV for the assessment of LRV stenosis is summarized in Table II. For LRV stenosis, agreement between DUS and DSV was weak and not statistically significant, with low concordance across stenosis grades (κ = 0.031; 95% confidence interval [CI], 0.117-0.178; *P* = .736). Agreement between dMRV and DSV was similarly weak and did not reach statistical significance (κ = 0.146; 95% CI, 0.080-0.373; *P* = .151). In contrast, direct comparison between DUS and dMRV demonstrated moderate agreement for LRV stenosis assessment, with statistically significant concordance (κ = 0.553; 95% CI, 0.333-0.772; *P* < .001).

**Table II:** Left renal vein (*LRV*) stenosis analysis

Agreement between DSV, DUS, and dMRV for LRV stenosis					
Method	Venography			Kappa (κ) concordance coefficient (95% CI)	*P* value
	<50%	50%-70%	>70%		
LRV	n = 36	n = 4			
DUS				0.031 (0.117-0.178)	.736
<50%	20 (55.6)	1 (25.0)			
50%-70%	14 (38.9)	1 (25.0)			
>70%	2 (5.6)	2 (50.0)			
dMRV				0.146 (0.080-0.373)	.151
<50%	24 (66.7)	1 (25.0)			
50%-70%	9 (25.0)	2 (50.0)			
>70%	3 (8.3)	1 (25.0)			

Agreement between DUS and dMRV for LRV stenosis					
dMRV	DUS			Kappa (κ) concordance coefficient (95 CI)	*P* value
	<50	50-70	>70		
LRV	n = 21	n = 15	n = 4	0.553 (0.333-0.772)	<.001
<50%	20 (95.2)	4 (26.7)	1 (25.0)		
50%-70%	0	9 (60.0)	2 (50.0)		
>70%	1 (4.8)	2 (13.3)	1 (25.0)		

*CI*, Confidence interval; *dMRV*, dynamic venous magnetic resonance angiography; *DSV*, dynamic subtraction venography; *DUS*, Doppler ultrasound.

Data are presented as number (%) unless otherwise indicated.

#### Analysis for left common iliac vein stenosis

Agreement between imaging modalities and DSV for the assessment of LCIV stenosis is summarized in Table III. For LCIV stenosis, moderate agreement was observed between DUS and DSV, with statistically significant concordance across stenosis grades (κ = 0.490; 95% CI, 0.212-0.178; *P* < .001). In contrast, agreement between dMRV and DSV was fair and did not reach statistical significance (κ = 0.211; 95% CI, 0.031-0.453; *P* = .056). Direct comparison between DUS and dMRV demonstrated fair agreement for LCIV stenosis assessment, with statistically significant concordance (κ = 0.331; 95% CI, 0.110-0.562; *P* = .003).

**Table III:** Left common iliac vein (LCIV) stenosis analysis

Agreement between DSV, DUS, and dMRV for LCIV stenosis					
Method	DSV			Kappa (κ) concordance coefficient (95% CI)	*P* value
	<50%	50%-70%	>70%		
LCIV	n = 17	n = 21	n = 2		
DUS				0.490 (0.212-0.178)	<.001
<50%	11 (64.7)	5 (23.8)	0		
50%-70%	6 (35.3)	16 (76.2)	0		
>70%	0	0	2 (100)		
dMRV				0.211 (0.031-0.453)	.056
<50%	10 (58.8)	6 (28.6)	0		
50%-70%	6 (35.3)	8 (38.1)	0		
>70%	1 (5.9)	7 (33.3)	2 (100)		

Agreement between DUS and dMRV for LCIV stenosis					
dMRV	DUS			Kappa (κ) concordance coefficient) (95% CI)	*P* value
	<50%	50%-70%	>70%		
LCIV	n = 16	n = 22	n = 2	0.331 (0.110-0.562)	.003
<50%	11 (68.8)	5 (22.7)	0		
50%-70%	4 (25.0)	10 (45.5)	0		
>70%	1 (6.3)	7 (3.8)	2 (100)		

*CI*, Confidence interval; *dMRV*, dynamic venous magnetic resonance angiography; *DSV*, digital subtraction venography; *DUS*, Doppler ultrasound.

Data are presented as number (%) unless otherwise indicated.

#### Analysis for gonadal reflux

Agreement between DUS, dMRV, and DSV for gonadal reflux assessment is summarized in Table IV. Very strong agreement was observed between dMRV and DSV (κ = 0.806; 95% CI, 0.590-1.022; *P* < .001), whereas DUS demonstrated moderate agreement with DSV (κ = 0.500; 95% CI, 0.170-0.830; *P* < .001). Direct comparison between DUS and dMRV showed moderate agreement for gonadal reflux assessment (κ = 0.453; 95% CI, 0.135-0.771; *P* = .001).

**Table IV:** Gonadal reflux analysis

Agreement between DSV, DUS, and dMRV for gonadal reflux				
Method	DSV		Kappa (κ) concordance coefficient (95% CI)	*P* value
	With reflux	Without reflux		
Gonadal reflux	n = 30	n = 10		
DUS			0.500 (0.170-0.830)	<.001
With reflux	30 (100)	6 (60.0)		
Without reflux	0	4 (40.0)		
dMRV			0.806 (0.590-1.022)	<.001
With reflux	28 (93.3)	1 (10.0)		
Without reflux	2 (6.7)	9 (90.0)		

Agreement between DUS and dMRV for gonadal reflux				
dMRV	DUS		Kappa (κ) concordance coefficient) (95 CI)	*P* value
	With reflux	Without reflux		
Gonadal reflux	n = 36	n = 4	0.453 (0.135-0.771)	.001
With reflux	29 (80.6)	0		
Without reflux	7 (19.4)	4 (100)		

*CI*, Confidence interval; *dMRV*, dynamic venous magnetic resonance angiography; *DSV*, digital subtraction venography; *DUS*, Doppler ultrasound.

Data are presented as number (%) unless otherwise indicated.

#### Analysis for internal iliac vein reflux

Agreement between DUS, dMRV, and DSV for iliac vein reflux assessment is summarized in Table V. For iliac vein reflux, agreement between DUS and DSV was weak and not statistically significant (κ = 0.124; 95% CI, 0.131-0.380; *P* = .334). Similarly, agreement between dMRV and DSV was fair but did not reach statistical significance (κ = 0.221; 95% CI, 0.028-0.471; *P* = .085). Direct comparison between DUS and dMRV showed fair agreement for iliac vein reflux assessment, without statistical significance (κ = 0.283; 95% CI, 0.070-0.637; *P* = .073).

**Table V:** Internal iliac vein reflux analysis

Agreement between DSV, DUS, and dMRV for internal iliac vein reflux				
Method	DSV		Kappa (κ) concordance coefficient (95% CI)	*P* value
	With reflux	Without reflux		
Internal iliac vein reflux	n = 21	n = 19		
DUS			0.124 (0.131-0.380)	.334
With reflux	6 (28.6)	3 (15.8)		
Without reflux	15 (71.4)	16 (84.2)		
dMRV			0.221 (0.028-0.471)	.085
With reflux	7 (33.3)	2 (10.5)		
Without reflux	14 (66.7)	17 (89.5)		

Agreement between DUS and dMRV for internal iliac vein reflux				
dMRV	DUS		Kappa (κ) concordance coefficient) (95 CI)	*P* value
	With reflux	Without reflux		
Internal iliac vein reflux	n = 9	n = 31	0.283 (0.070-0.637)	.073
With reflux	4 (44.4)	5 (16.1)		
Without reflux	5 (55.6)	26 (83.9)		

*CI*, Confidence interval; *dMRV*, dynamic venous magnetic resonance angiography; *DSV*, digital subtraction venography; *DUS*, Doppler ultrasound.

Data are presented as number (%) unless otherwise indicated.

#### Diagnostic accuracy between modalities

The diagnostic accuracy of each method for detecting disease (stenosis ≥50% and/or presence of reflux) is presented in Table VI, including sensitivity, specificity, positive predictive value, negative predictive value, and percentage of correct classifications. For gonadal reflux, DUS demonstrated a sensitivity of 100%, whereas dMRV showed a sensitivity of 93.3%. Although sensitivity for iliac vein involvement was low, both methods exhibited good specificity. Accuracy metrics for each venous territory and imaging modality are illustrated in Supplementary Figs 1-4 (online only).

**Table VI:** Diagnostic accuracy of Doppler ultrasound (*DUS*) and dynamic venous magnetic resonance angiography (*dMRV*) for gonadal and iliac vein reflux

Method	Sensitivity	Specificity	Positive predictive value	Negative predictive value	Accuracy rate
LRV					
DUS	75.0	58.3	16.7	95.5	60.0
dMRV	75.0	72.2	23.1	96.3	72.5
LCIV					
DUS	78.3	58.8	72.0	66.7	70.0
dMRV	78.3	64.7	75.0	68.8	72.5
Gonadal reflux					
DUS	100	40.0	83.3	100	85.0
dMRV	93.3	90.0	96.6	81.8	92.5
Iliac vein reflux					
DUS	28.6	84.2	66.7	51.6	55.0
dMRV	33.3	89.5	77.8	54.8	60.0

*LCIV*, Left common iliac vein; *LRV*, left renal vein.

Data are presented as percentages.

## Discussion

This cross-sectional study evaluated 40 women with severe symptoms compatible with PeVD, including chronic pelvic pain, dyspareunia, and dysmenorrhea, with overall symptom evolution longer than 6 months and worsening in the orthostatic position, resulting in significant limitation of daily activities. Patients were referred from other services after prior transvaginal ultrasound suggesting PeVD and were assessed at a dedicated pelvic congestion outpatient clinic. All patients underwent venous DUS, dMRV, and pelvic DSV. Using DSV as the reference standard, we investigated the agreement and diagnostic accuracy of DUS and dMRV for detecting venous reflux and stenosis in different pelvic venous territories.

Regarding LRV stenosis, we found no significant agreement between either DUS or dMRV and DSV. This result underscores the superior accuracy of DSV for assessing LRV compression in the context of suspected nutcracker physiology. Anatomic depth, respiratory motion, variable hemodynamics, and technical limitations of both ultrasound and dMRV may contribute to the difficulty in reliably grading the degree of renal vein stenosis with noninvasive imaging alone.^11^^,^^12^ These findings suggest that, when LRV compression is a major diagnostic concern, DSV remains essential to confirm the diagnosis and to guide potential endovascular treatment.

With respect to LCIV stenosis consistent with May–Thurner syndrome, we observed moderate agreement between DUS and DSV, whereas dMRV did not show meaningful concordance. Similar limitations of dMRV have been reported in prior studies, which demonstrated limited reliability for grading iliac vein stenosis when compared with invasive reference standards, including low specificity, a tendency to overestimate luminal narrowing, and inadequate reflection of hemodynamic significance.^13^

When comparing DUS and dMRV directly, we observed moderate agreement between DUS and dMRV for LRV stenosis, but only fair agreement for LCIV stenosis. Taken together, these findings still suggest that DUS and dMRV are generally comparable in the diagnostic work-up of PeVD, while also indicating that dMRV may add value when a more detailed anatomical characterization is required—particularly in scenarios where DUS results are uncertain or when iliac involvement is clinically relevant.^14^^,^^15^

In contrast, when evaluating gonadal vein reflux, which represents one of the main mechanisms associated with pelvic varicosities and pelvic vein disorders, both DUS and dMRV showed substantial to excellent agreement with DSV, with dMRV achieving a very strong level of concordance. Importantly, gonadal vein reflux represents the most consistent hemodynamic abnormality associated with symptomatic pelvic venous congestion, whereas isolated venous compressions, such as LCIV or LRV stenosis, may be insufficient to produce pelvic congestion symptoms in the absence of reflux.^3^^,^^16^ This suggests that noninvasive imaging modalities, particularly dMRV, can accurately identify gonadal vein reflux and may be sufficient to establish the diagnosis in many patients when clinical presentation and imaging findings are concordant. These results are consistent with recent evidence emphasizing gonadal vein reflux as the primary pathophysiological mechanism underlying pelvic venous disorders and demonstrating high diagnostic performance of noninvasive imaging, especially dMRV, when compared with invasive DSV.^17^

When comparing DUS and dMRV directly, we found moderate agreement for gonadal vein reflux. Overall, this supports the idea that both modalities are broadly comparable for PeVD evaluation, especially for detecting reflux. Given that DUS is widely accessible and relatively low-cost, it can serve as an initial screening modality and a practical tool to support treatment planning in many clinical settings. dMRV may be particularly useful when a comprehensive anatomical overview of the pelvic venous system is needed or when DUS findings are inconclusive.

We also evaluated the diagnostic accuracy of each method for detecting disease, defined as ≥50% stenosis and/or the presence of reflux in the evaluated venous segment. For gonadal reflux, DUS demonstrated a sensitivity of 100%, whereas dMRV showed a sensitivity of 93.3%, with both methods maintaining good specificity. Although sensitivity for iliac vein involvement was low for both techniques, specificity remained high, suggesting that positive findings in this territory are likely to be true positives, whereas negative examinations should be interpreted with caution.

The heterogeneous diagnostic performance observed across different venous territories in the present study reflects the complex and multifactorial nature of pelvic vein disorders. Previous systematic reviews have shown that the accuracy of noninvasive imaging modalities varies substantially according to the venous segment evaluated and the underlying pathophysiological mechanism, with consistently better performance for reflux-dominant patterns than for compressive syndromes.^18^

This study has several limitations. Its retrospective design and the relatively small, highly selected sample from a single tertiary referral center limit the generalizability of the findings and support the interpretation of this work as a pilot study. In addition, the low number of patients with severe stenosis limits the robustness of subgroup analyses and precludes definitive conclusions for this subgroup. Furthermore, due to the retrospective nature of the study and limitations of the electronic medical records, data on comorbid generalized anxiety disorder or major depressive disorder, polycystic ovary syndrome, and mean duration of pelvic pain were not consistently available and therefore could not be included in the analysis. All examinations were performed in a specialized pelvic congestion clinic by experienced operators, which may not reflect the performance of DUS or dMRV in less specialized settings. In addition, DSV was performed intraoperatively under optimal conditions and interpreted in real time, whereas DUS and dMRV were obtained as part of routine preoperative assessment and are inherently more operator- and protocol-dependent. This difference in acquisition context may have contributed to the higher level of agreement observed for DSV-based assessments and may partly explain the lower concordance of noninvasive modalities in certain venous territories. Moreover, all duplex ultrasound examinations were performed with patients in the supine position, which may have underestimated venous reflux, particularly within the internal iliac vein tributaries, and may partly explain the only moderate agreement observed for hypogastric reflux. Because of the retrospective design, ultrasound diagnostic criteria remained unchanged throughout the study period, precluding iterative refinement based on imaging–DSV correlation. We did not assess interobserver variability, and we did not correlate imaging findings with treatment response or long-term clinical outcomes. Future prospective studies with larger, more heterogeneous populations and standardized imaging protocols are needed to validate our results and to refine the diagnostic algorithm for PeVD.

Despite these limitations, our findings support an important role for noninvasive imaging in the workup of PeVD. DUS and dMRV showed good performance for detecting gonadal vein reflux and acceptable agreement for some stenotic lesions, whereas DSV remains indispensable for comprehensive hemodynamic assessment and for complex or equivocal cases. A stepwise diagnostic approach—using DUS as the initial test, followed by dMRV or DSV when indicated—may optimize diagnostic accuracy while minimizing procedural risk in patients with suspected PeVD.

Finally, in the context of PeVD, compressive syndromes such as May–Thurner and Nutcracker are not consistently associated with the clinical presentation of PeVD and, when present in isolation, may be insufficient to explain typical symptoms. In contrast, ovarian vein reflux represents the most consistent and clinically relevant hemodynamic finding associated with symptomatic PeVD.^19^ Given the high level of agreement observed between DUS and DSV for the detection of gonadal reflux, DUS alone may be sufficient as an initial diagnostic modality in most cases. However, when treatment planning is indicated, additional imaging techniques, including computed tomography angiography, dMRV, or invasive DSV, may be required to provide detailed anatomic characterization and guide therapeutic decision-making. In selected patients, a judicious use of noninvasive imaging may reduce the need for invasive DSV, reserving it primarily for complex cases or when endovascular intervention is planned.

## Conclusions

In this cohort of women with suspected pelvic congestion syndrome, DUS and dMRV showed good performance for detecting gonadal vein reflux and moderate agreement for selected stenotic lesions compared with DSV. Although neither modality reliably assessed renal vein stenosis, both contributed meaningful diagnostic information. DUS serves as an accessible first-line test, whereas dMRV is valuable when further anatomic detail is needed. DSV remains essential for definitive hemodynamic evaluation and treatment planning. These findings support a stepwise diagnostic approach that prioritizes noninvasive imaging and reserves DSV for complex or inconclusive cases.

## Author Contributions

Conception and design: MC, JB, VB, WJ, MM, AZ, PL, AA, NdL

Analysis and interpretation: MC, LD, JB, VB, WJ, MM, AZ, PL, AA, NdL

Data collection: MC, JB, AA, NdL

Writing the article: MC, LD, JB, VB, WJ, MM, AZ, PL, AA, NdL

Critical revision of the article: MC, LD, JB, VB, WJ, MM, AZ, PL, AA, NdL

Final approval of the article: MC, LD, JB, VB, WJ, MM, AZ, PL, AA, NdL

Statistical analysis: MC, LD, JB

Obtained funding: Not applicable

Overall responsibility: MC

## Funding

None.

## Disclosures

Disclosures M.L.C. is enrolled as a student in the Postgraduate Program in Anesthesiology, Surgical Sciences, and Perioperative Medicine at the Faculty of Medicine, University of São Paulo, São Paulo, Brazil, under the supervision of Antonio Eduardo Zerati. The remaining authors report no conflicts.

## References

[1] Bookwalter C.A., VanBuren W.M., Neisen M.J., Bjarnason H. (2019). Imaging appearance and nonsurgical management of pelvic venous congestion syndrome. Radiographics.

[2] Wu W.C., Hsu W.H., Chang T.C., Huang L.W. (2025). Pelvic congestion syndrome due to central venous outflow obstruction: a single-center experience with may-Thurner and nutcracker syndromes. Int J Gynaecol Obstet.

[3] Meissner M.H., Khilnani N.M., Labropoulos N. (2021). The Symptoms-Varices-Pathophysiology classification of pelvic venous disorders: a report of the American Vein & Lymphatic Society International Working Group on Pelvic Venous Disorders. J Vasc Surg Venous Lymphatic Disord.

[4] Phillips D., Deipolyi A.R., Hesketh R.L., Midia M., Oklu R. (2014). Pelvic congestion syndrome: etiology of pain, diagnosis, and clinical management. J Vasc Interv Radiol.

[5] Borghi C., Dell’Atti L. (2016). Pelvic congestion syndrome: the current state of the literature. Arch Gynecol Obstet.

[6] Steenbeek M.P., van der Vleuten C.J.M., Schultze Kool L.J., Nieboer T.E. (2018). Noninvasive diagnostic tools for pelvic congestion syndrome: a systematic review. Acta Obstet Gynecol Scand.

[7] Osman A.M., Mordi A., Khattab R. (2021). Female pelvic congestion syndrome: how can CT and MRI help in the management decision?. Br J Radiol.

[8] Gloviczki P., Comerota A.J., Dalsing M.C. (2011). The care of patients with varicose veins and associated chronic venous diseases: clinical practice guidelines of the Society for Vascular Surgery and the American Venous Forum. J Vasc Surg.

[9] Yang W., Sun L., Shi Y., Ye T., Li Q. (2025). Radiological evaluation of pelvic venous disorders: a comprehensive review. Eur J Radiol.

[10] Landis J.R., Koch G.G. (1977). The measurement of observer agreement for categorical data. Biometrics.

[11] Park S.J., Lim J.W., Ko Y.T. (2004). Diagnosis of pelvic congestion syndrome using transabdominal and transvaginal sonography. AJR Am J Roentgenol.

[12] Al-Katib S., Shetty M., Jafri S.M.A., Jafri S.Z.H. (2017). Radiologic assessment of native renal vasculature: a multimodality review. Radiographics.

[13] Saleem T., Lucas M., Raju S. (2022). Comparison of intravascular ultrasound and magnetic resonance venography in the diagnosis of chronic iliac venous disease. J Vasc Surg Venous Lymphat Disord.

[14] Szkodziak F., Woźniak S., Kudła M. (2025). The usefulness of transvaginal ultrasonography in the diagnosis of pelvic venous disorders. Sci Rep.

[15] Benacerraf B.R., Abuhamad A.Z., Bromley B. (2015). Consider ultrasound first for imaging the female pelvis. Am J Obstet Gynecol.

[16] Hansrani V., Riding D., Seif M.W. (2023). Pelvic vein incompetence and chronic pelvic pain: a case-control study. BJOG.

[17] Shahat M., Abdelbaqy O.M.A., AbdelHakam A.M., Ali S.H., Attalla K. (2024). Can cross-sectional imaging replace diagnostic venography in pelvic venous disorder (PeVD)?. J Vasc Surg Venous Lymphat Disord.

[18] Behzadi A.H., Khilnani N.M., Zhang W. (2019). Pelvic cardiovascular magnetic resonance venography: venous changes with patient position and hydration status. J Cardiovasc Magn Reson.

[19] Barge T.F., Uberoi R. (2022). Symptomatic pelvic venous insufficiency: a review of the current controversies in pathophysiology, diagnosis, and management. Clin Radiol.

