# Supplementary material for: Lesion-specific comparison of pelvic venous ultrasound, dynamic magnetic resonance venography, and digital subtraction venography in pelvic vein disorders
Source: J Vasc Surg Venous Lymphat Disord. 2026 May 8;14(5):102517. doi: 10.1016/j.jvsv.2026.102517 (PMC13241635; doi:10.1016/j.jvsv.2026.102517)

**SUPPLEMENTARY MATERIAL**

**TITLE:** Lesion-specific comparison of pelvic venous ultrasound, dynamic magnetic resonance venography and digital subtraction venography in pelvic vein disorders

**Table I.** Diagnostic criteria for each imaging modality.

**Figure 1.** Sensitivity, specificity, positive predictive value, and negative predictive value of Doppler ultrasound and dMRV for LRV (left renal vein) involvement.

**Figure 2.** Sensitivity, specificity, positive predictive value, and negative predictive value of DUS and dMRV for LCIV (left common iliac vein).

**Figure 3.** Sensitivity, specificity, positive predictive value, and negative predictive value of DUS and dMRV for gonadal vein reflux.

**Figure 4.** Sensitivity, specificity, positive predictive value, and negative predictive value of DUS and dMRV for iliac vein reflux.

**Table 1.** Diagnostic criteria for each imaging modality.

| **Venous territory** | **Degree / Feature** | **DUS** | **dMRV** | **DRV** |
| --- | --- | --- | --- | --- |
| LRV stenosis | <50% | Peak velocity ratio < 2.0 between stenotic and pre-stenotic segments | Cross-sectional area reduction <50% compared with pre-stenotic segment | Mild caliber reduction without significant pressure gradient |
|  | 50–70% | Peak velocity ratio 2.0–2.5 | Cross-sectional area reduction 50–70% | Moderate caliber reduction, delayed washout |
|  | >70% | Peak velocity ratio > 2.5 | Cross-sectional area reduction >70% | Severe compression with delayed washout and LRV–IVC gradient ≥ 3 mmHg |
| LCIV stenosis | <50% | Peak velocity and diameter ratios compatible with <50% stenosis | Cross-sectional area reduction <50% | Mild extrinsic compression without relevant collaterals |
|  | 50–70% | Peak velocity and diameter ratios compatible with 50–70% stenosis | Cross-sectional area reduction 50–70% | Moderate compression with pre-stenotic dilatation and/or collaterals |
|  | >70% | Peak velocity and diameter ratios compatible with >70% stenosis | Cross-sectional area reduction >70% | Severe compression with extensive collateral circulation and delayed washout |
| Gonadal reflux | Present | Retrograde flow in gonadal vein and ISOOAI pathways | Retrograde opacification of gonadal vein and pelvic venous plexuses on dynamic sequences | Retrograde filling of gonadal vein and pelvic varices during contrast injection |
| Iliac (hypogastric) reflux | Present | Retrograde flow in internal iliac vein tributaries (ISOOAI pathways) | Retrograde flow from internal iliac tributaries into pelvic venous plexuses | Retrograde opacification of internal iliac vein branches |
| DRV: digital subtraction venography – DUS – Duplex Ultrasonography; dMRV – Dynamic Magnetic Resonance Venography; LRV – Left Renal Vein; LCIV – Left Common Iliac Vein; IVC – Inferior Vena Cava; ISOOAI – Insufficiency of the Superficial/Ovarian and Obstructive/Axial Iliac veins | | | | |

**Figure 1.** Sensitivity, specificity, positive predictive value, and negative predictive value of Doppler ultrasound and dMRV for LRV (left renal vein) involvement.


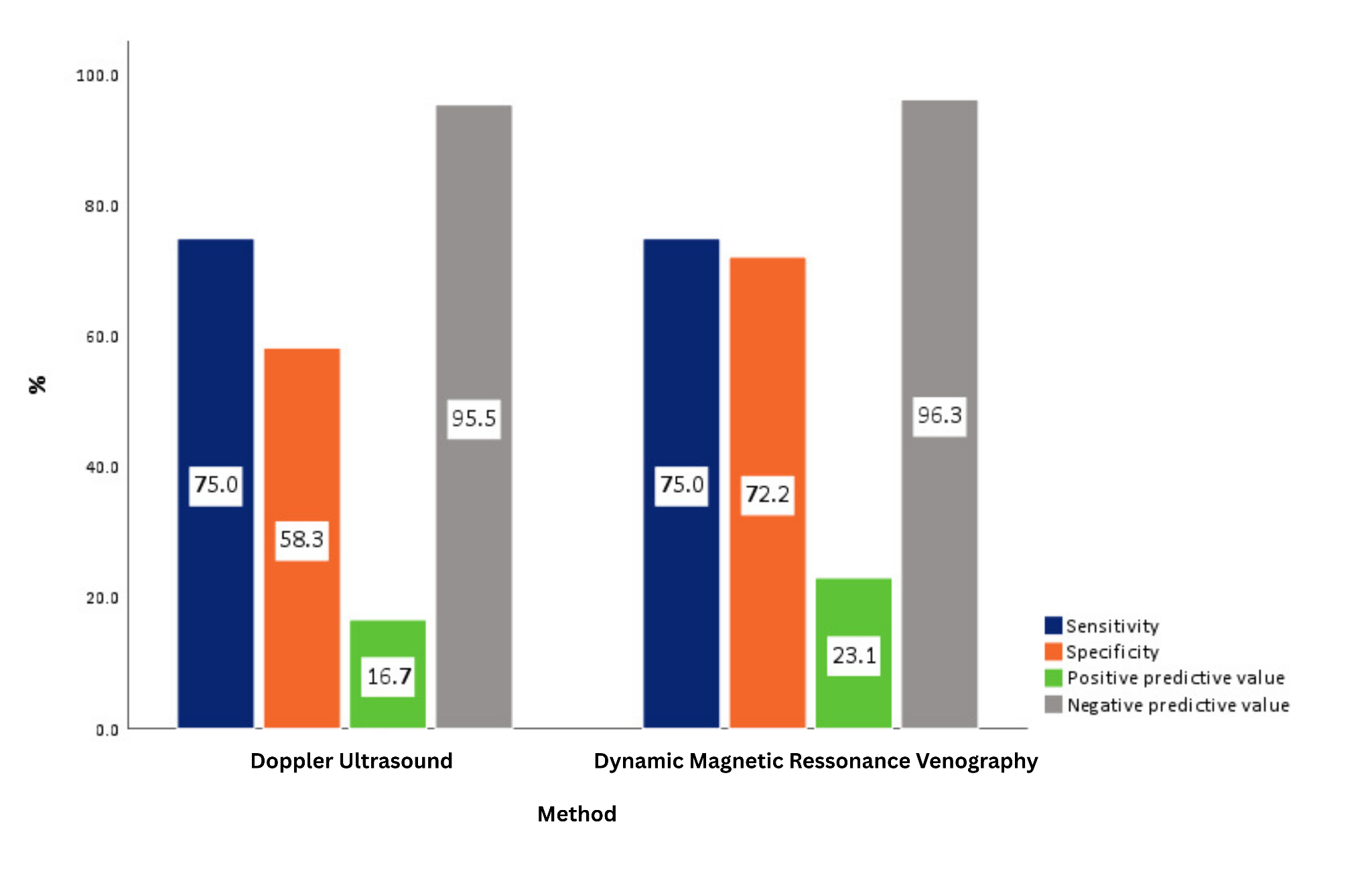


**Figure 2.** Sensitivity, specificity, positive predictive value, and negative predictive value of DUS and dMRV for LCIV (left common iliac vein).


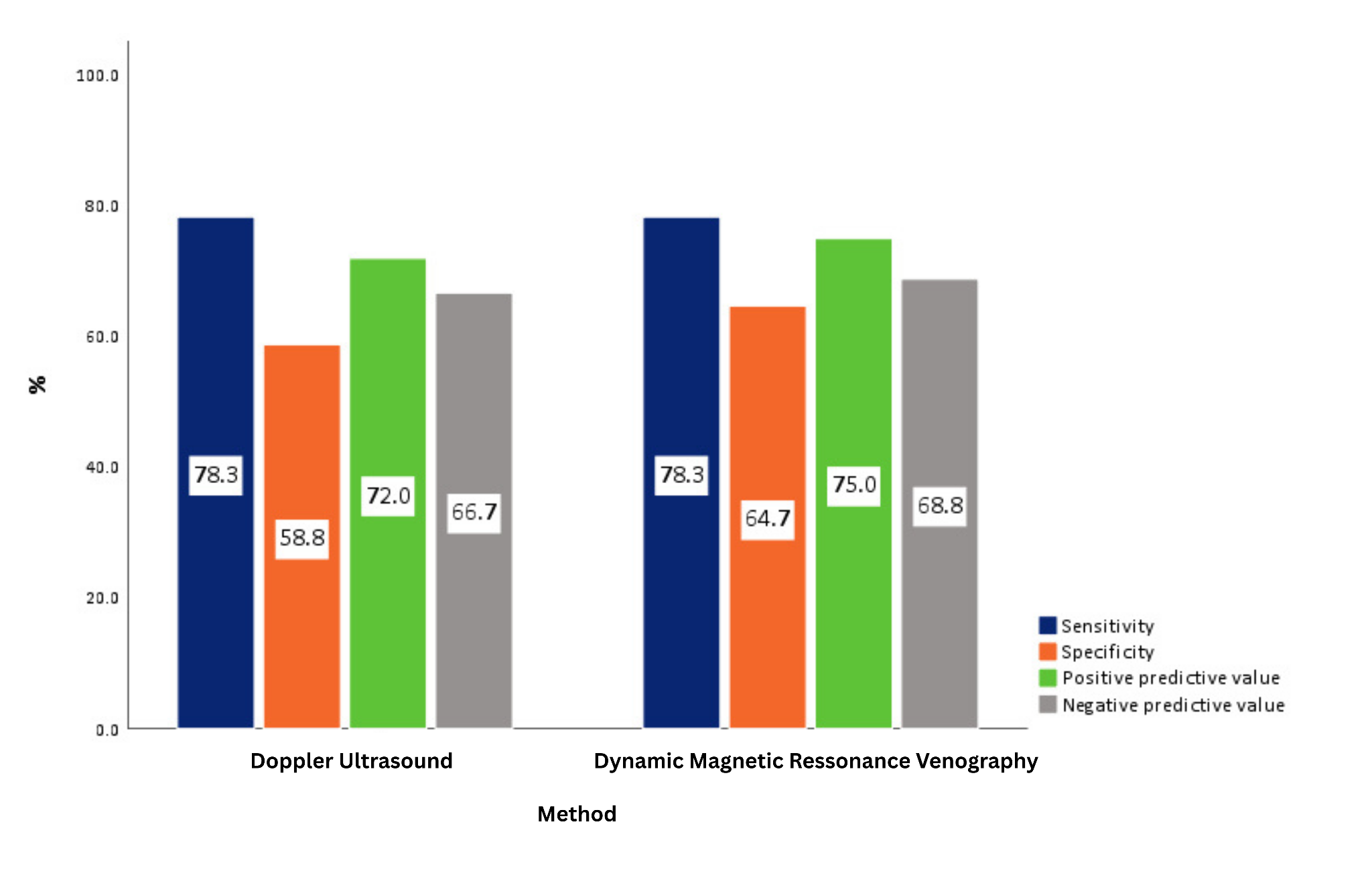


**Figure 3.** Sensitivity, specificity, positive predictive value, and negative predictive value of DUS and dMRV for gonadal vein reflux.


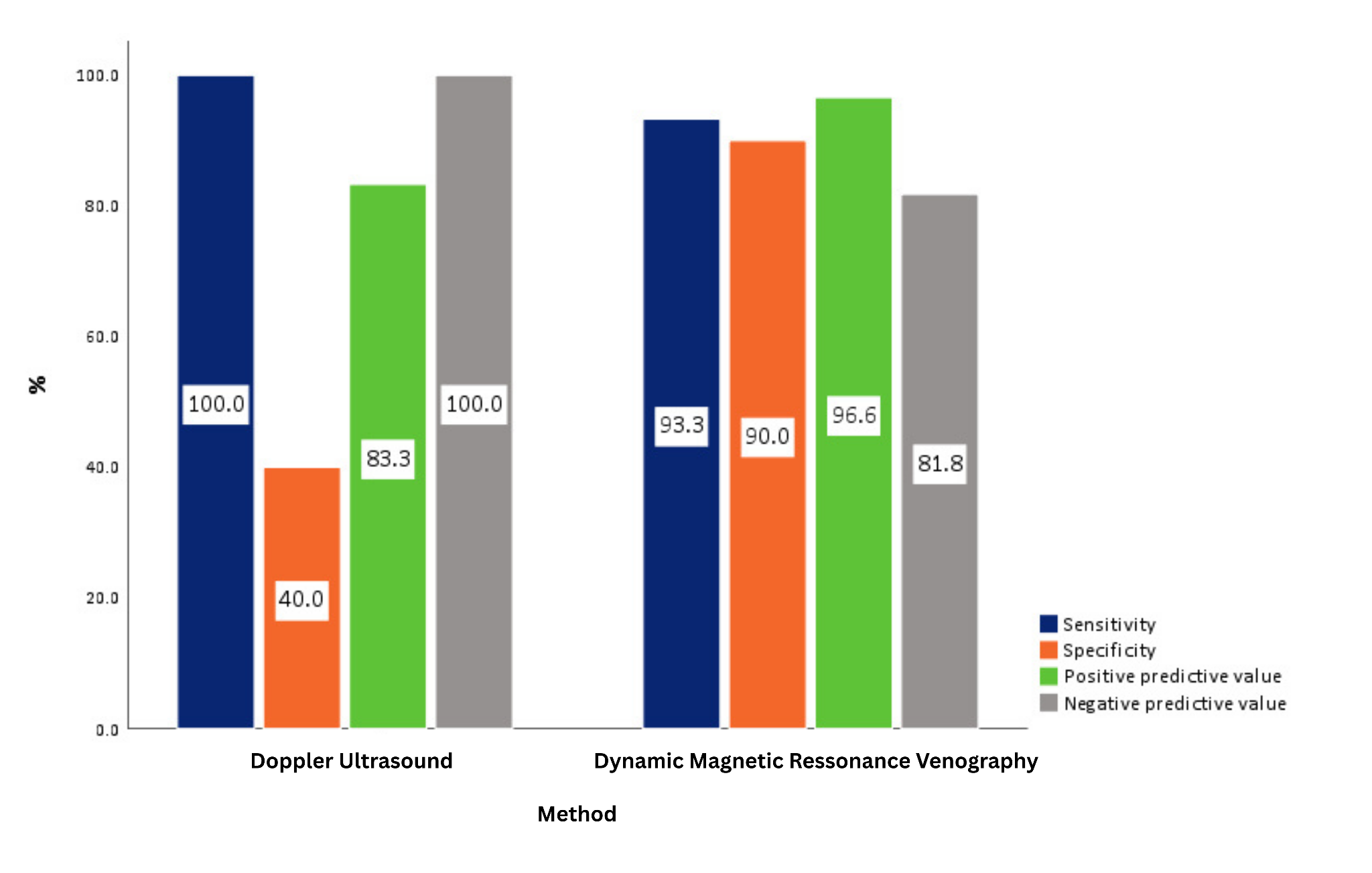


**Figure 4.** Sensitivity, specificity, positive predictive value, and negative predictive value of DUS and dMRV for iliac vein reflux.


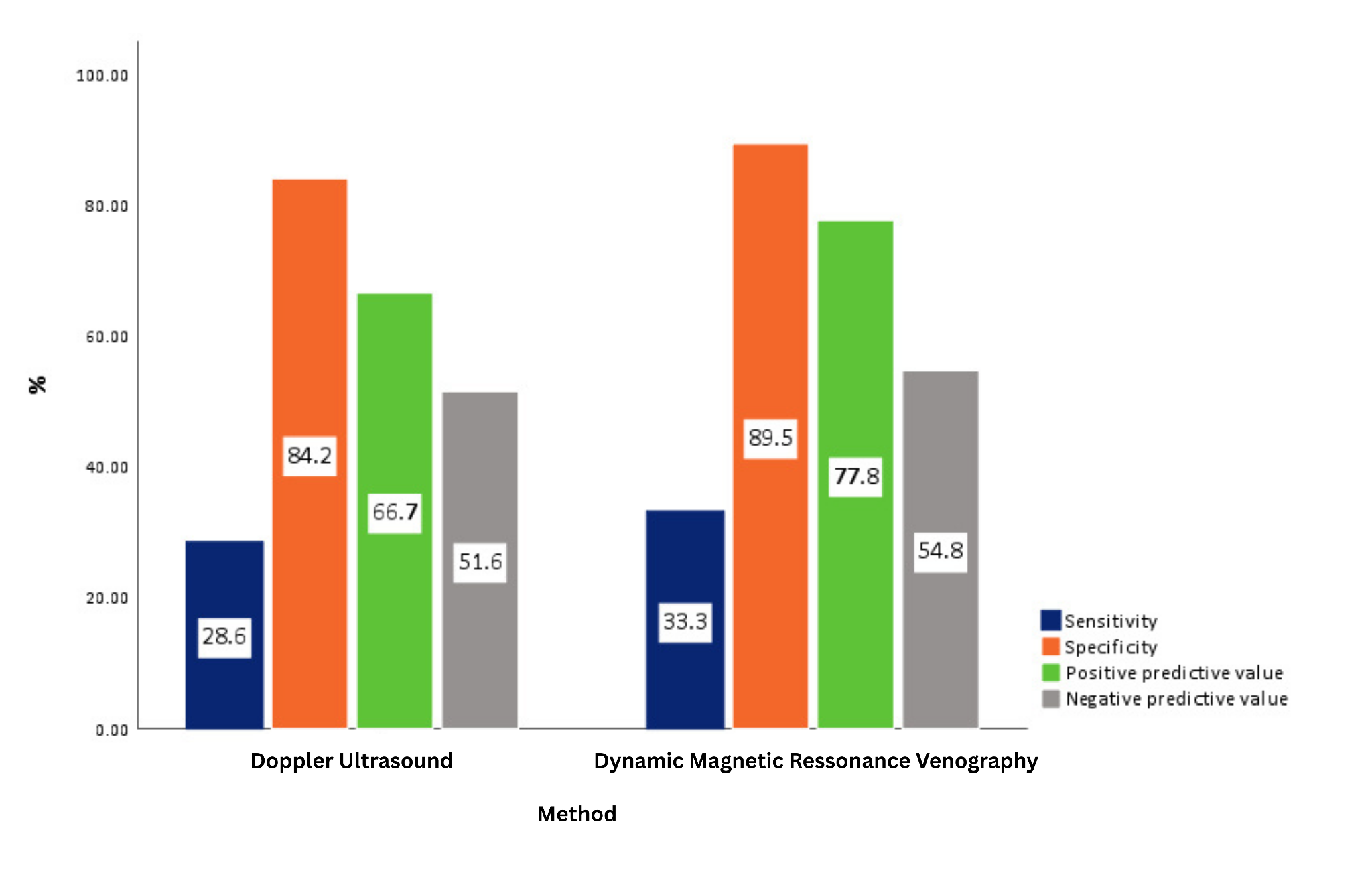

Supplement: Supplementary Material [file mmc1.docx]
